# Nanodroplets versus Nanofibers Ion-Selective Optodes toward Biocompatible Sensors

**DOI:** 10.1021/acsomega.5c03974

**Published:** 2025-06-27

**Authors:** Anna Konefał, Justyna Kalisz, Emilia Stelmach, Piotr Piątek, Krzysztof Maksymiuk, Agata Michalska

**Affiliations:** † Faculty of Chemistry, University of Warsaw, Pasteura 1, 02-093 Warsaw, Poland; ‡ Lukasiewicz Research Network, Industrial Chemistry Institute, Rydygiera 8, 01-793 Warsaw, Poland

## Abstract

This work compared the performance of ion-selective optodes in different nanostructural formats: nanodroplets and nanofibers prepared from biocompatible materials using classical ionophores and ion exchangers. Nanodroplets tested contained minute amounts of plasticizer acetyl tributyl citrate stabilized with poly­(vinyl alcohol) and were suspended in the aqueous phase. It was shown that this system was not directly transferable to the nanofiber format; thus, nanofibers obtained from polycaprolactone were used as the support material. Both nanodroplets and nanofibers were used in Nile blue/Nile red-based systems operating as novel ion-selective sensor optical transducers, allowing sensors to be applied in unbuffered samples. As a model system, calcium sensors were prepared and tested. It was shown that, regardless of format, optodes were characterized by increased emission intensity at the Nile red characteristic wavelength (605 nm) for increasing calcium ion concentration in the sample. Nile blue emission changes for varying analyte concentrations were specific for the format (they changed for nanodroplets, whereas for nanofibers, they were similar within the range of experimental error). For both systems, a linear dependence of the ratio of emission intensity was recorded for Nile red and Nile blue characteristic wavelength on the changes in analyte concentration in the sample within the range from 10^–5^ to 10^–1^ M. The unique advantages of using nanofiber mats include the determination of optical signals for both sensors present in solution and probes removed from solution; moreover, the same sensor can be transferred between different solutions. This is a clear advantage of the application of nanofiber mats as sensors, especially when used on a dedicated, 3D-made holder.

## Graphical abstract

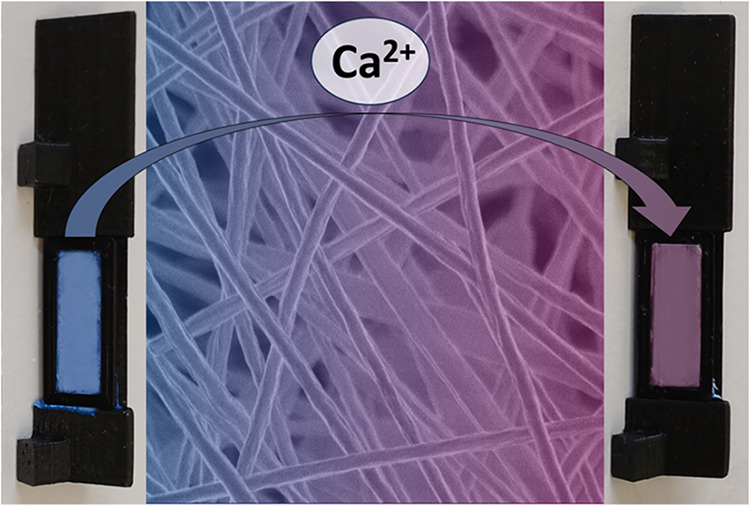

## Introduction

Originally, ion-selective optode films contained lipophilic ionophores, ion exchangers, and optical transducers (as most of the ionophores are not optically active) embedded within a lipophilic matrix: polymer, optionally plasticized.^1^ Similarly, as other ion-selective sensors, optodes are typically based on the application of PVC together with the DOS or oNPOE plasticizer; this clearly raises issues related to the safety and biocompatibility of probes, regardless of their format.

The classical optode concept requires the application of a chromoionophore and a pH-sensitive dye as an optical transducer. The response mechanism involves incorporation of analyte cations into the probe, due to selective binding by the ionophore, and requires expulsion of hydrogen ions to maintain the electroneutrality of the probe. To record optical signals dependent on analyte concentration changes in solution, the sample pH has to be kept constant and close to the p*K*_a_ of the dye.^2–4^ This clear disadvantage of classical systems renders practical application and has stimulated the search for alternative mechanisms. Among different solutions, the application of polarity sensitive dyes, typically offering a decrease in the signal for increasing analyte concentration (turn off sensors),^4^ was proposed. Alternatively, conducting polymers^2^ or, more recently, Nile blue/Nile red (NB/NR) systems,^3^ both offering a turn-on mode of responses, have been applied. In this respect, the Nile blue/Nile red system seems to be highly attractive, as it allows for recording the linear dependence of the ratio of emission intensity of two dyes involved within a broad analyte concentration range.^3,5^ In this system, the receptor contains an ionophore, a cation exchanger, and an adjunctive, mobile cationic reagent, which is expelled from the bulk (to maintain the probe electroneutrality) in response to analyte cation binding.^3,5^ As an adjunctive (mobile) cation, Nile blue was applied. This choice is motivated by the properties of the dye: Nile blue cations are relatively hydrophilic (dye is water-soluble), and the properties of NB are pH-independent in a broad range. Additionally, an unobvious advantage of using Nile blue is that it is a commercially available salt also containing Nile red dye, which is spontaneously generated from Nile blue.^3,5^ NR in the presence of Nile blue cations is optically silent; however, release of Nile blue cations to solution (due to incorporation of analyte cations) results in the bright emission of NR.

The crucial aspect of using nanostructural optical probes is the format of the sensors. The significant improvement in terms of shortening the response time of sensors was achieved when polymeric films were replaced by micro- and nanospheres.^6,7^ The nanostructural optodes proposed were in fact a suspension of minute droplets of a lipophilic plasticizer (originally used in film systems) containing an ionophore and an ion exchanger, stabilized by a polymer (e.g., Pluronic) or cross-linked polymeric nanostructures (polymaleic anhydride).^8,9^

It should be stressed that nanoparticle or micelle suspension-type probes resemble reagents rather than sensors, and their application is prone to permanently contaminate the sample.^10^ Thus, there is still a challenge in the field to prepare truly nanostructural sensors, to benefit from the advantages of nanostructural character, as well as being a real sensor that can be introduced and removed from the sample when needed. Toward this end, the application of nanofibers seems to be attractive. Early examples of the application of nanofibers as optodes focused on monitoring local pH changes were based on the application of just a pH-sensitive dye alone in the polymer poly­(ε-caprolactone) (PCL) or poly­(lactic-*co*-glycolic acid) matrix.^11^ Nanofibers were also used as the support—the surface of inert polymer nanofibers was modified with an ultrathin liquid layer of plasticizer solution of an ionophore, an ion exchanger, and a pH-sensitive dye optical transducer (the classical optode system).^12^ The other issue potentially affecting the application of nanofiber mats as optodes is the wettability of the nanofiber structure.^13^

The aim of this work was to compare the performance of nanodroplet- and nanofiber-based optodes to investigate the possibility of preparation of nanostructural optodes based on a biocompatible polymer/plasticizer mixture, using a classical ionophore and an ion exchanger but benefiting from a Nile blue/Nile red system as a pH-independent optical transducer, allowing the application of sensors in the absence of a pH buffer.^3^ This approach in principle is a step toward further application of nanofiber-based sensors as wearable sensors, due to minimization of the toxic effects of the most abundant materials used. Thus, optodes were prepared using poly­(vinyl alcohol) (PVA) and/or poly­(ε-caprolactone), known as a biocompatible material. The lipophilic liquid (plasticizer) used was acetyl tributyl citrate (ATBC). As a model system, calcium-selective nanofiber mat optodes have been prepared and tested. The issues related to the translation of a successful analytical system of nanoparticles to the nanofiber format are highlighted.

## Experimental Section

### Reagents

Poly­(vinyl alcohol) (PVA) 31 or 130 kDa, polycaprolactone (PCL) 80 kDa, Nile blue chloride (NBCl), potassium tetrakis­(4-chlorophenyl)­borate (KTChPB), calcium-selective ionophore (ETH 1001), acetyl tributyl citrate (ATBC), tris­(hydroxymethyl)­aminomethane (Tris), and dimethylformamide (DMF) were obtained from Sigma-Aldrich. All of the chemicals used, including dyes, were reagent grade, except for the ionophore and ion exchanger, which were selectophore grade.

Tetrahydrofuran (THF) was obtained from POCh (Gliwice, Poland).

Doubly distilled and freshly deionized water (resistance of 18.2 MΩ cm, Milli-Q Plus, Millipore, Austria) was used throughout this work.

### Apparatus

Nanofibers were obtained using an electrospinning apparatus that consisted of a DC power supply (ELSR30P300, Technix), a syringe with a stainless steel needle (0.2 mm; 27G), an infusion pump (KDS 100, *K*_d_ Scientific), and a collector plate with aluminum foil.

Nanofibers were cross-linked in a furnace at 160 °C (ThermConcept, Germany).

The morphology of nanofibers was studied using scanning electron microscopy (SEM, Merlin FE-SEM, Zeiss, Germany). Prior to the measurements, nanofiber mats were coated with a thin gold–palladium layer. Nanofiber diameter measurements were carried out from SEM images using the image processing program Fiji/ImageJ.

The water contact angle was measured for the nanofibrous mat by using a contact angle apparatus with a digital camera (Delta Optical, Poland). Water contact angle of the mats was calculated based on an image as an average of 3 values obtained using the ImageJ program.

The obtained nanodroplets were characterized using Zetasizer Nano ZS Malvern (scattering angle 173 degrees).

All fluorimetric experiments were performed by using a spectrofluorimeter (Varian, Cary Eclipse). After excitation at a wavelength of 550 nm, emission intensity was recorded within the range of 570 to 800 nm. The slits used were 10 nm, and the detector voltage was maintained in the range from 450 to 600 V.

### Preparation of Ion-Selective Nanofiber Mats with a Cocktail Solution

Two types of nanofiber mats were chosen to immobilize PVA nanodroplets: cross-linked PVA and PCL; both polymers were biocompatible.

To prepare the PCL mat, a 12% PCL solution was dissolved in a THF/DMF mixture in a volume ratio of 1:1 using a magnetic stirrer and heated to 60 °C for 18 h. The solution was added to a syringe, which was placed in a syringe pump. A high-voltage source was connected to a needle and set at 14 kV. The polymer suspension flow rate was set at 1 mL/h. Fiber mats were collected for 1.5 h on an electrically grounded collector (aluminum foil). The distance between the needle and the collector was 15 cm. The collector was rotated at a speed of 100 rpm. Electrospinning was carried out at 21 °C with a humidity of 35% controlled by a dehydrator and air conditioning. The obtained nanofiber mats were left in the laboratory atmosphere overnight.

A PVA mat was prepared by thermal cross-linking. First, 435 mg of PVA (130 kDa) and 50 mg of citric acid (cross-linking agent) were dissolved in 5 mL of water using a magnetic stirrer and heated to 65 °C for 6 h. Then, the solution was added to a syringe, which was placed in an infusion pump. The high-voltage source was connected to a needle and was set at 15 kV. The polymer suspension flow rate was controlled by a syringe pump and was set at 0.65 mL/h. Fiber mats were collected for 4 h on an electrically grounded collector (aluminum foil). The distance between the needle and the collector was equal to 15 cm. Electrospinning was carried out at 21 °C with a humidity of 40% controlled by a dehydrator and air conditioning. The obtained nanofiber mats were left in a laboratory atmosphere overnight. Then, nanofibers were cross-linked for 1 h.

Ca^2+^-selective solution (cocktail) was prepared: 0.5 mg of NBCl, 0.6 mg of KTChPB, 4.4 mg of ETH 1001, and 10 mg of ATBC were dissolved in 1 mL of propan-2-ol under magnetic stirring for 20 min. To prepare nanodroplets, 500 μL of the cocktail solution was added to 50 mL of 0.5% PVA (31 kDa) solution, and the mixture was stirred for 30 min with an open vial.

To be able to transfer the nanofiber mat-based sensor among different samples, as well as to ensure reproducibility of measurements (in particular, the optical readout of the sensor removed and then again placed in the sample solution), a holder was designed and prepared using a 3D printing approach. The design of the holder took into account easy mounting/dismounting of the nanofiber mat in the holder and easy but reproducible placing of the mat (in the holder) in the cuvette in the optical path. Additional requirements were to be able to add reagents to the cuvette. The developed design and photo of the prepared holder immersed in the cuvette are presented in Figure 1A.

**Figure 1:**
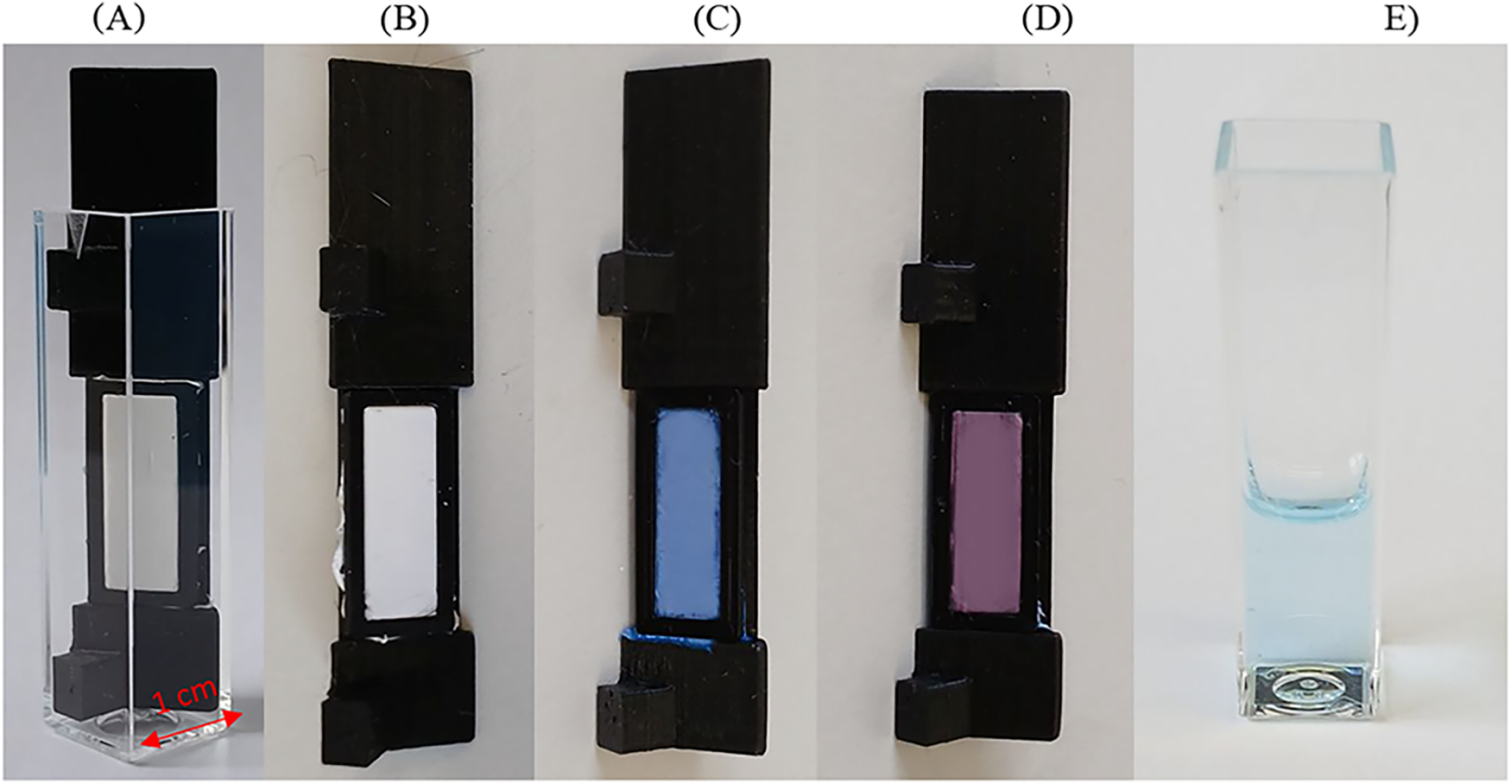
Images of the prepared 3D holder with the nanofiber mat (A) in the cuvette and (B) the holder with the immobilized PCL mat; the holder with the PCL mat with cocktail before (C) and after (D) contact with solution of 10^–1^ M CaCl_2_ and (E) solution of 10^–1^ M CaCl_2_ after 30 min contact with the mat.

A rectangular shape of appropriate size (adapted to the holder) was cut out from ready-made PCL or PVA cross-linked mats. A fragment of the mat was immobilized using a 3D printed holder^14^ and placed in a measuring cuvette (Figure 1B). The proposed system allows for the placement of a mat portion in the cuvette with an angle of incidence of the exciting beam equal to 35°. On each immobilized mat with PCL nanofibers on the holder, 40 μL of the cocktail mixture (500 μL of Ca^2+^-selective cocktail +500 μL of 5% PVA (31 kDa) solution) was drop-cast (Figure 1C). However, on PVA cross-linked mats in the holder, 40 μL of the Ca^2+^-selective solution (cocktail) was applied. After 15 min, the holder with a fragment of the nanofiber mat was placed in a cuvette. Then, aliquots of stock CaCl_2_ solution (or other electrolyte salts) were added to obtain the desired concentration of Ca^2+^ ions, and spectra were recorded.

## Results and Discussion

### Nanodroplets

Surprisingly, to the best of our knowledge, the PVA polymer was rarely considered a coalescence-preventing agent for liquid nanodroplet-type optodes. Therefore, a set of experiments were performed to verify the performance of ATBC containing an ionophore, an ion exchanger, and optical transducer nanodroplets stabilized with PVA as nano-optodes.

As-obtained PVA-stabilized nanodroplets in DI water were characterized with a zeta potential of −26.1 mV, clearly suggesting the stability of the obtained nanosphere dispersion. The mean diameter of the obtained nanospheres was close to 260 ± 36 nm (*n* = 7).

Emission spectra recorded for nanospheres containing solution (in unbuffered aqueous samples), in the absence of calcium ions in the solution, showed a single emission maximum at 670 nm, as reported previously for PCL sphere optodes based on the Nile blue/Nile red optical transducer system^3^ (Figure 2A). A similar emission spectrum, but with increased intensity, was observed for 10^–6^ M CaCl_2_ solution. The increase of calcium ion concentration in the sample, to reach 10^–5^ M concentration, resulted in some increase of emission intensity in the whole wavelength range, especially at ca. 670 nm, i.e., corresponding to Nile blue emission. This behavior is similar to that observed for polymeric spheres,^3^ yet the emission intensity increase is significantly more pronounced. It should be stressed that for nanoprobes tested in this work, already at a concentration of analyte of 10^–4^ M, a significant increase of emission intensity ascribed to Nile red, i.e., at 605 nm, was observed, whereas the Nile blue emission was not affected (within the range of experimental error). A further increase of Ca^2+^ ion concentration in solution resulted in a gradual increase of emission intensity observed at 605 nm. For 10^–2^ and 10^–1^ M CaCl_2_ present in solution, some decrease in the intensity of Nile blue emission was observed. The dependence of emission ratio read at 605 to 670 nm, Nile red to Nile blue, Figure 2A inset, was linearly dependent on the changes of the logarithm of analyte concentration in solution within the concentration range from 10^–5^ to 0.1 M (*R* ^2^ = 0.986). A significant difference was observed when it comes to the change of the zeta potential of herein proposed nano-optodes, as compared to polymeric spheres described previously.^3^ For PVA-stabilized nanodroplets, the initial increase of Ca^2+^ concentration results in a vast change in zeta potential from −24.1 mV in 10^–6^ M solution to values close to −1 mV for analyte concentration higher than 10^–3^ M. Thus, the zeta potential increase is achieved for relatively narrow concentration change, as compared to polymeric spheres. Moreover, the observed change of zeta potential corresponds well to the initial increase of the emission intensity at the Nile blue characteristic wavelength observed for the change in concentration from 10^–6^ to 10^–5^ M (Figure 2B). This suggests that the zeta potential change is related to the release of Nile blue cations from the bulk of the nanodroplets; the process is naturally much faster for the liquid core of the probes described here than for polymeric structures used previously.^3^ This contributes to a more pronounced increase of Nile red emission intensity and ultimately to the higher sensitivity of Nile red emission changes for analyte concentration changes in solution. This effect is a unique advantage of using a liquid core of applied nanoprobes. The facile release of Nile blue cations probably results in a decrease of emission intensity observed at the Nile blue characteristic wavelength for concentrations of analyte higher than 10^–5^ M (Figure 2).

**Figure 2:**
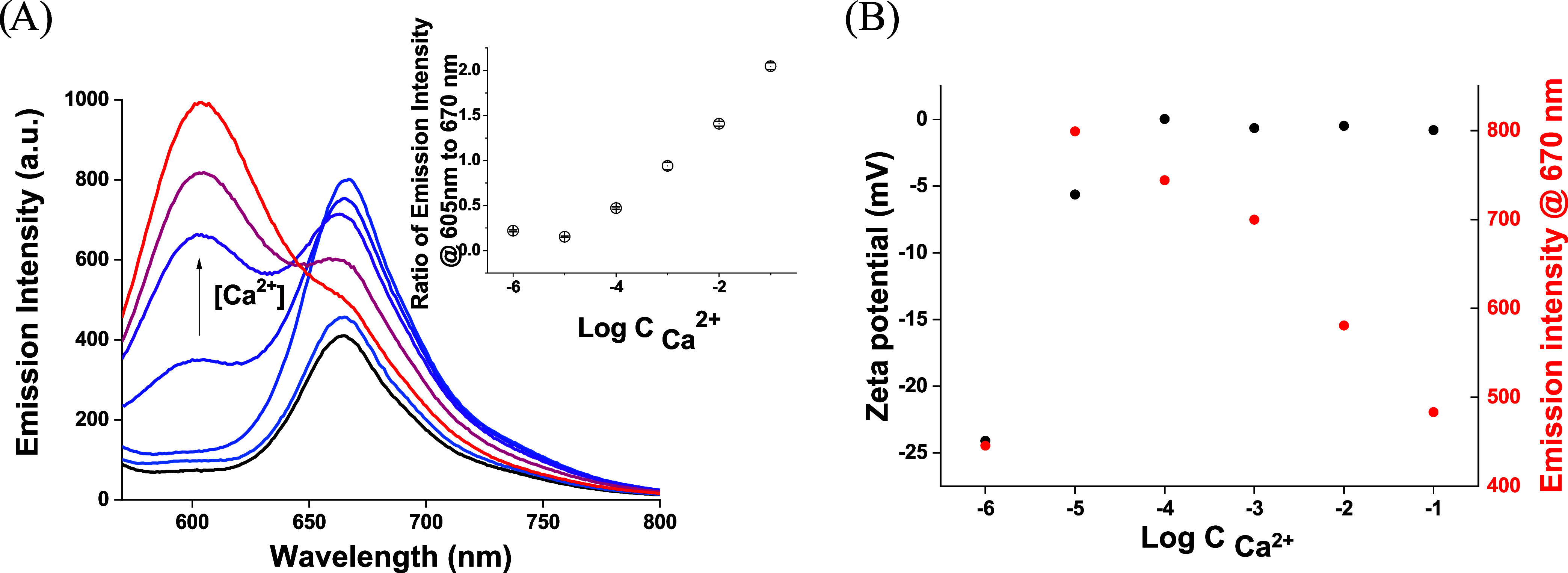
(A) Emission spectra of Ca^2+^-selective nano-optodes (black line) in the absence of an analyte and for increasing concentration of Ca^2+^, within the range from 10^–6^ to 10^–1^ M, recorded in unbuffered aqueous solution after 5 min probe–sample contact time and an excitation wavelength of 550 nm. Inset: dependence of the ratio of emission intensity read at 605 to 670 nm on the logarithm of Ca^2+^ ion concentration in solution ± SD (*n* = 3). (B) Dependence of zeta potential and the emission intensity at Nile blue on the Ca^2+^ ion concentration from 10^–6^ to 10^–1^ M.

As expected, PVA-based probes were highly selective toward model interferents Mg^2+^, Na^+^, and K^+^ ions present in solution in concentrations within the range from 10^–5^ to 0.1 M. In the interfering cation-containing solutions, a single emission peak at 670 nm, ascribed to Nile blue, was recorded, and the emission intensity recorded was not affected by the change of interferent concentration in solution within the range of experimental error (results not shown). Consequently, the ratio of emission intensities recorded at 605 to 670 nm was constant and independent of interferent ions concentrations in these solutions.

Similar dependences of signals on ion concentration in solution were obtained for PVA-based nanodroplets when tested in Tris buffer (0.1 M, pH = 7.5) in both calcium ions and interfering ion solutions (Figure S1A). The emission intensity ratio (650 to 670 nm) calculated for spectra recorded in CaCl_2_ solutions was linearly dependent on the logarithm of concentration within the range from 10^–5^ to 0.1 M (*R* ^2^ = 0.990). The emission intensity ratio obtained for the interferent solution was not (within the range of experimental error) affected by changes in these ion concentrations (results not shown). On the other hand, responses recorded for calcium ion solution in the background of 10^–3^ M HCl resulted in a linear dependence of the emission intensity ratio (650 to 670 nm) covering the concentration range from 10^–6^ to 0.1 M (*R* ^2^ = 0.995). This clearly shows the applicability of the herein used optical transducer system also in the low pH range, i.e., in the range inaccessible for classical systems based on chromoionophores (Figure S1B).

### Nanofiber Mats

The results presented above clearly show that PVA-stabilized ion-selective nano-optodes offer attractive analytical properties. The brief comparison of the surface area of the nanofiber mat portion to the polymeric film of equal diameters offers a 3 orders of magnitude larger surface area. Taking into account the relatively small diameter of the nanofibers, this contributes to a significant increase in the surface-to-volume ratio compared to other systems.^12^ This has an important beneficial effect on sensors, requiring ion exchange of analyte ions on the interface between the probe and sample, and ion transport through the bulk of the probe to generate analytical signals.^12^

Inspired by the analytical performance of PVA-stabilized nanodroplets presented above, we aimed to prepare sensors that can be transferred among different solutions: PVA or PCL nanofiber mats modified by PVA nanodroplets, and test/compare the performance of these systems in comparison to nanodroplets.

The PVA polymer is characterized by water dispersibility; this property allows application of PVA as a dispersion stabilizing agent for nano- and microparticles used as ion-selective optical sensors,^1,7^ including those described above. Water dispersion of PVA was also used as a feeding solution to prepare the nanofibers. However, to use PVA nanofibers as sensor polymers, cross-linking is required to decrease solubility in the aqueous phase. For the sake of biocompatibility of obtained systems, Flores-Arriaga and Stone’s method^15,16^ based on the application of citric acid was preferred. The PVA cross-linking process is typically performed at ca. 65 °C. The scheme of the esterification reaction is shown in Figure S2. To minimize the risk of the adverse effect of applied temperature on the ionophore/ion exchanger, an ion-selective cocktail solution in an ATBC biocompatible plasticizer was applied on the nanofiber mats after immobilizing them on a 3D holder.

The SEM image of the as-obtained PVA nanofibers in electrospinning is presented in Figure S3. PVA nanofibers are relatively thin, and the mean diameter is close to 52.3 ± 8.2 nm. In the course of cross-linking, the diameter of nanofibers was not significantly affected; after the process, it was equal to 60.4 ± 13.2 nm (Figure S3). The images obtained post application of the ATBC-based ion-selective cocktail (ionophore, ion exchanger, and Nile blue) clearly suggest that PVA nanofibers are coated by a (liquid) layer. In contrast to systems studied previously (PVDF coated by a DOS plasticizer-based cocktail),^12^ the liquid layer was not well adsorbed within the cross-linked PVA nanofiber surface. The liquid layer was easy to be removed/transferred to other substrates in both solution and dry states, just by touching. This effect can be explained by the high hydrophobicity of cross-linked PVA (water contact angle measured for the cross-linked nanofiber mat was equal to 50° ± 5°) and the relatively high lipophilicity of the ATBC-based cocktail. As a result, the optical responses of the obtained structures are highly irreproducible (both in the absence and presence of analyte in solution; results not shown).

Clearly, another (biocompatible) polymeric support was required; thus, PCL nanofibers were prepared and tested. However, PCL nanofiber mats are characterized by a very high water contact angle, Figure S4A, equal to 136° ± 1°, making this material difficult to use as a sensor intended to operate in the aqueous phase. To improve the wettability of the PCL mat and to introduce an ion-selective cocktail to the phase, a PVA-stabilized ATBC nanodroplet suspension was applied on the nanofiber structure. This resulted in a significant decrease in water contact angle to 45° (Figure S4B). The SEM image of as-obtained PCL nanofibers presented in Figure S4A shows that fibers are characterized by a diameter close to 245 ± 43 nm. After the application of PVA-based ATBC and cocktail-loaded nanodroplets, the nanofiber diameter increases slightly to reach 278 ± 54 nm (Figure S4B). Nanofibers, as shown in the SEM image, are slightly bent and expanded. This effect is ascribed to the incorporation of an ATBC-based liquid into the PCL structures.

Emission spectra recorded for this system in the absence of the analyte, calcium ions, in solution (unbuffered aqueous samples were used), Figure 3, are significantly different from those obtained for nanodroplet dispersion (Figure 2). Two emission maxima were observed: higher at 605 nm, ascribed to Nile red, and lower at 670 nm, i.e., at Nile blue characteristic wavelength. This clearly suggests that Nile blue is less effectively incorporated into the bulk of nanofibers compared to Nile red. As a result, in the absence of analyte, Nile red within the nanofibers (probe) is less quenched (compared to the PVA nanodroplet suspension). As a result, the emission of this dye has already been observed in the absence of an analyte. An increase in calcium ion contents in the sample resulted in increased emission at the Nile red characteristic wavelength, with a minimum effect on emission at 670 nm, i.e., Nile blue characteristic wavelength (Figure 3). The increase in the emission is also visible to the naked eye by changing the color of the mat from blue to pink (Figure 1C,D). The dependence of the emission intensity ratio read at 605 to 670 nm, Nile red to Nile blue, on the logarithm of concentration, Figure 3, was linear within the concentration range from 10^–5^ to 0.1 M (*R* ^2^ = 0.995). The longer contact time of the nanofiber mat with the sample resulted in higher emission intensities recorded and ultimately in the higher sensitivity of the emission ratio (read at 605 to 670 nm) versus the logarithm of concentration changes, without affecting the linear response range (for the same experimental parameters; Figure 3B). This effect can be related to the release of Nile blue from the nanofiber bulk or its surface to the sample solution. The release from the surface of nanofibers seems more probable, as initially (in the absence of analyte), significant Nile red emission was observed. Indeed, emission spectra recorded for solutions, after removing the nanofiber mat in a holder (Figure S5), clearly show some Nile blue emission, which is also visible to the naked eye (Figure 1E). This effect can be expected considering the results obtained for nanodroplets of ATBC stabilized by PVA; when tested in water, both emission and zeta potential change. It should be stressed, however, that the emission intensities observed are relatively small, compared to the magnitude of emission signals recorded for the nanofiber mat (using the same experimental parameters; Figure 3). Thus, the compilation of results shown in Figures 3 and S5 clearly shows that the effect of the release of Nile blue from the nanofiber mats does not adversely affect the performance of the sensor. It should also be added that in the case of classical optodes, ion sensing also leads to the release of ions (in that case, H^+^) to the sample (typically buffer).

**Figure 3:**
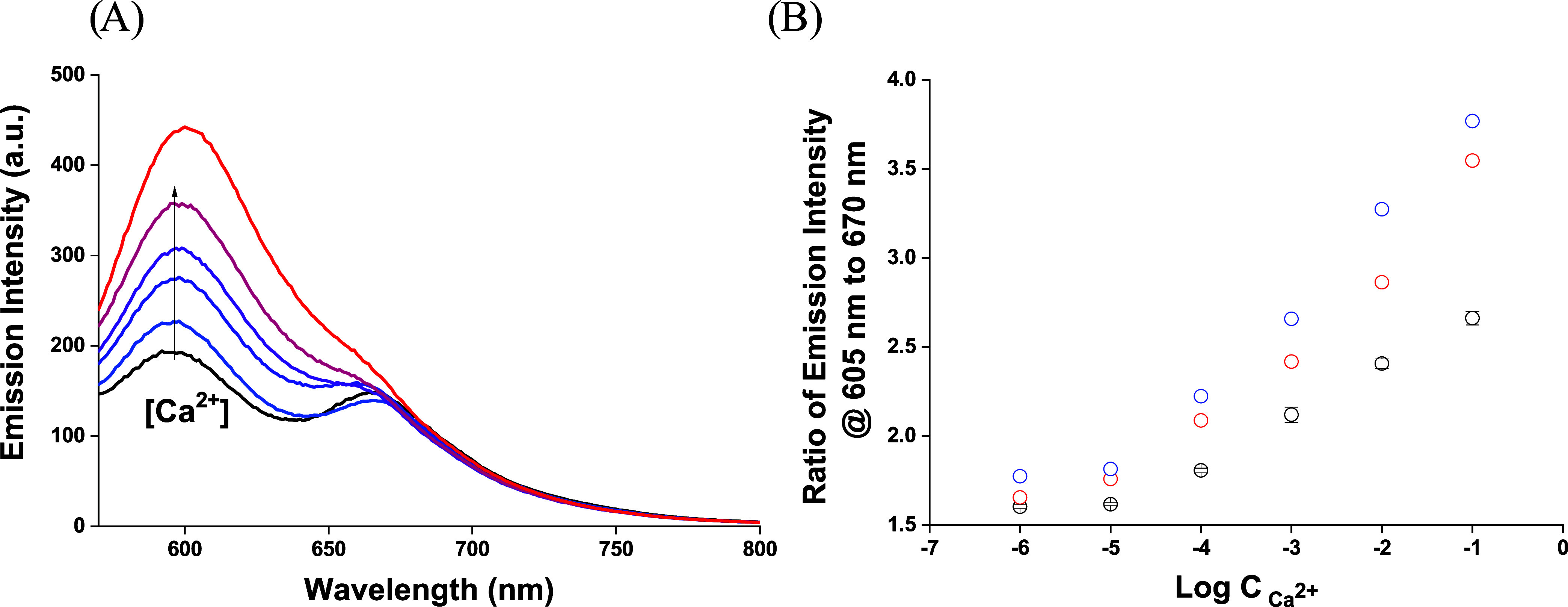
(A) Emission spectra of the Ca^2+^-selective PCL mat for increasing concentration of Ca^2+^, within the range from 10^–6^ to 10^–1^ M, recorded in an unbuffered aqueous sample after 5 min probe–sample contact time and an excitation wavelength of 550 nm. (B) Dependence of the ratio of emission intensity read at 605 to 670 nm on the logarithm of Ca^2+^ ion concentration in solution; (black circle) after 5 min contact time (mean ± SD, *n* = 3) and after 30 min time recorded for (red circle) mat and sample solution and (blue circle) mat removed from the sample solution.

Using a nanofiber mat in a holder allowed the comparison of responses obtained for probes immersed in solution and free-standing after removal from the sample. Similar responses were obtained for the nanofiber mat removed from the sample solution and then tested (placed in another empty cuvette; Figure 3). In the presence of model interferents tested, magnesium, sodium, or potassium ion (Figure S6) emission spectra recorded within the concentration range from 10^–6^ to 0.1 M (within the range of experimental error) were not affected by the changes in electrolyte type or concentration in solution, proving the high selectivity of this system assured due to the presence of a highly selective ionophore.

To further prove the applicability of the herein presented sensor approach, we aimed to test the responses of one sensor in different concentrations of the analyte, as typically done for, e.g., ion-selective electrodes. In this experiment, a mat placed in a holder was transferred in sequence from 10^–5^ to 0.1 M CaCl_2_ solutions; the results obtained for 3 different sensors tested in parallel are shown in Figure 4. The results presented in Figure 4 clearly demonstrate that the mat placed in the holder allows high sensor-to-sensor reproducibility; moreover, the response pattern obtained is also reproducible; similar signals were obtained for different sensors tested. The clear advantage of using a nanofiber mat compared to micelle suspension is related to the possibility of the easy removal of the sensor from the sample.

**Figure 4:**
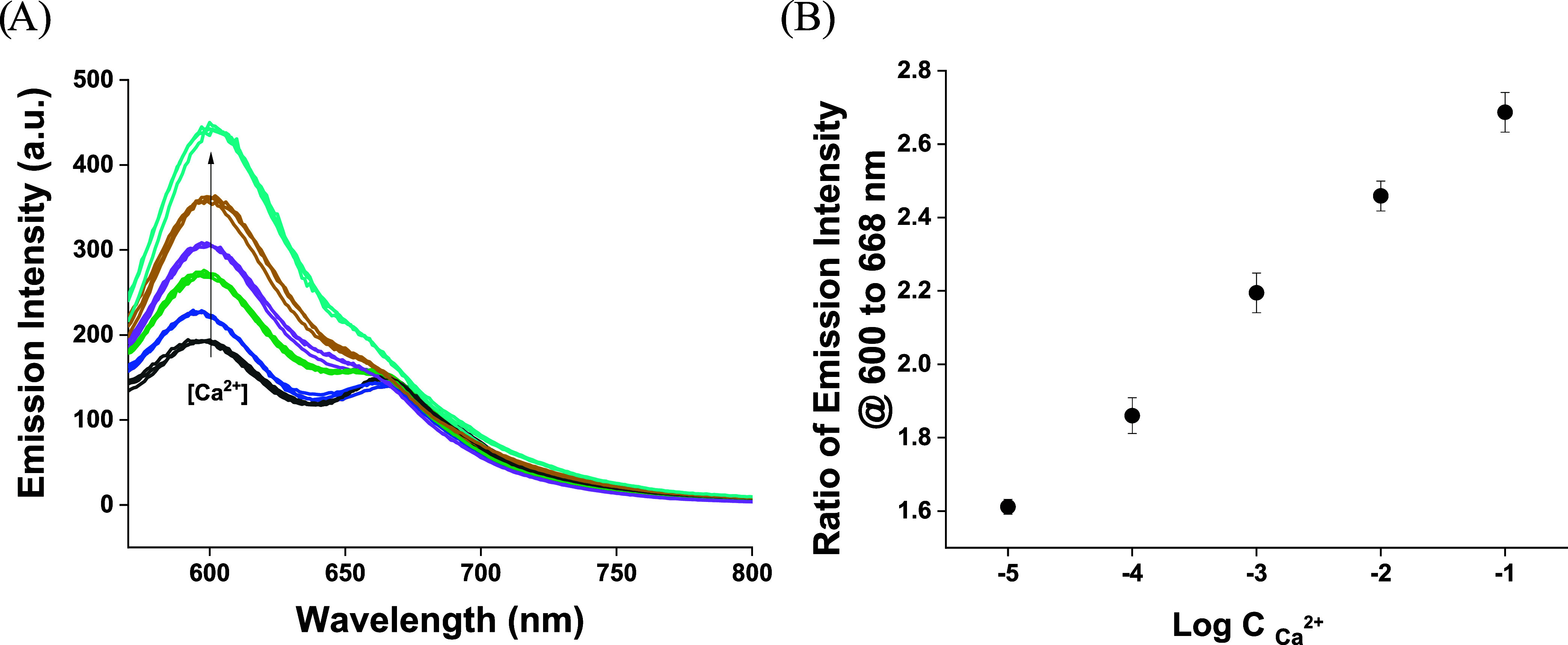
(A) Emission spectra of 3 different sensors: Ca^2+^-selective PCL mat for increasing concentration of Ca^2+^, within the range from 10^–5^ to 10^–1^ M; the sensor was transferred from solution of low concentration to a that of high concentration, and responses were recorded in an unbuffered aqueous sample after 5 min probe–sample contact time and an excitation wavelength of 550 nm. (B) Dependence of the ratio of emission intensity read at 605 to 670 nm on the logarithm of Ca^2+^ ion concentration in solution (mean obtained for 3 different sensors ± SD, *n* = 3).

## Conclusions

Ion-selective optodes using a Nile blue/Nile red system as an optical transducer were prepared in nanodroplet and nanofiber formats, using biocompatible polymers and plasticizers. The performance of sensors in different formats was compared. Regardless of sensor format, incorporation of analyte ions into the probe led to an increase in emission intensity at the Nile red characteristic wavelength (605 nm). Due to the presence of Nile blue in the system, the ratio of emission intensity recorded for Nile red and Nile blue can be used as the analytical signal. The proposed system can be used in the absence of a buffer and also in acidic samples. The emission ratio obtained was linearly dependent on the change in the logarithm of analyte concentration within the concentration range from 10^–5^ to 10^–1^ M, for both systems studied. The application of nanofiber mats seems advantageous, as it allows easy transfer of the probe, placed in a special holder, between different samples. Nanofiber mats placed in a holder offer increased reproducibility of optical signals. Similar signals were obtained for the system in contact with the solution as well as removed from the solution.

## Supplementary Material



## Notes

Emission spectra of Ca^2+^-selective nano-optodes for increasing concentration of Ca^2+^; mechanism of the esterification reaction occurring during cross-linking of PVA; SEM images and size distribution of PVA nanofibers; water contact angles of PVA nanofibers; SEM images of the as-obtained PCL nanofiber mat and water contact angle; emission spectra of solution post removing the Ca^2+^-selective PCL mat holder, recorded for different analyte Ca^2+^ concentration; and emission spectra of the Ca^2+^-selective PCL mat for increasing concentration of model interferents (PDF)
