# Supplementary material for: Nanodroplets versus Nanofibers Ion-Selective Optodes toward Biocompatible Sensors
Source: ACS Omega. 2025 Jun 27;10(26):28429–35. doi: 10.1021/acsomega.5c03974 (PMC12242670; doi:10.1021/acsomega.5c03974)
Supplement: Supplementary file 1 [file ao5c03974_si_001.pdf]

Supporting information

For

Nanodroplets vs nanofibers ion-selective optodes, towards biocompatible sensors

Anna Konefał[1,2], Justyna Kalisz[1], Emilia Stelmach[1], Piotr Piątek[1], Krzysztof Maksymiuk[1],

Agata Michalska[1]

[1] Faculty of Chemistry, University of Warsaw, Pasteura 1, 02-093 Warsaw, Poland
* agatam@chem.uw.edu.pl, +48 22 55 26 331

[2] Lukasiewicz Research Network, Industrial Chemistry Institute, Rydygiera 8, 01-793 Warsaw, Poland

Table of contents

1. Emission spectra of Ca^{2+}-selective nano-optodes for increasing concentration of Ca^{2+}.

2. Mechanism of esterification reaction occurring during crosslinking of PVA.

3. SEM images and size distribution of PVA nanofibers.

4. Water contact angles of PVA nanofibers.

5. SEM images of as obtained PCL nanofibers.

6. PCL nanofibers mat and water contact angle.

7. Emission spectra of solution post removing Ca^{2+}-selective PCL mat holder, recorded for different analyte Ca^{2+} concentration.

8. Emission spectra of Ca^{2+}-selective PCL mat for increasing concentration of model interferents.

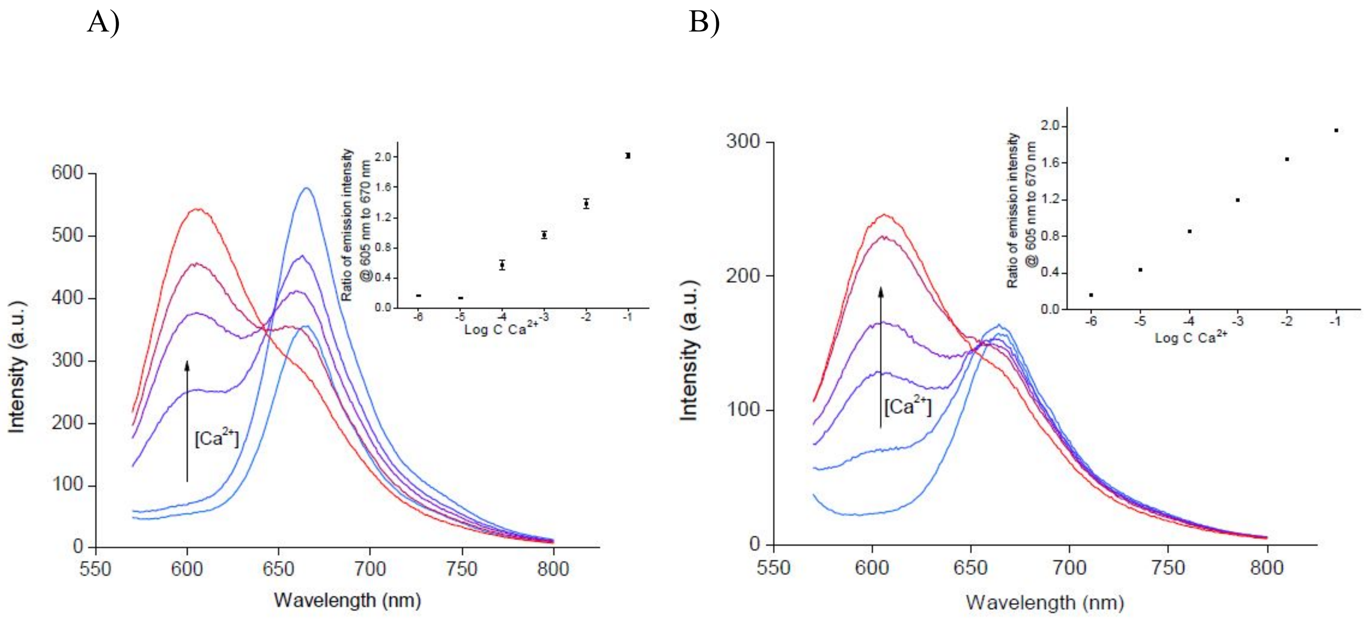

Fig. S1. Emission spectra of Ca^{2+}-selective nano-optodes for increasing concentration of Ca^{2+}, within the range from 10^{-6} to 10^{-1} M, recorded in A) Tris buffered samples (0.1 M, pH = 7.5) and B) in 10^{-3} M HCl after 5 min probe – sample contact time, excitation wavelength 550 nm. Inset: dependence of ratio of emission intensity read at 605 nm to 670 nm on logarithm of Ca^{2+} ions concentration in solution, for figure B ± SD (n = 3).

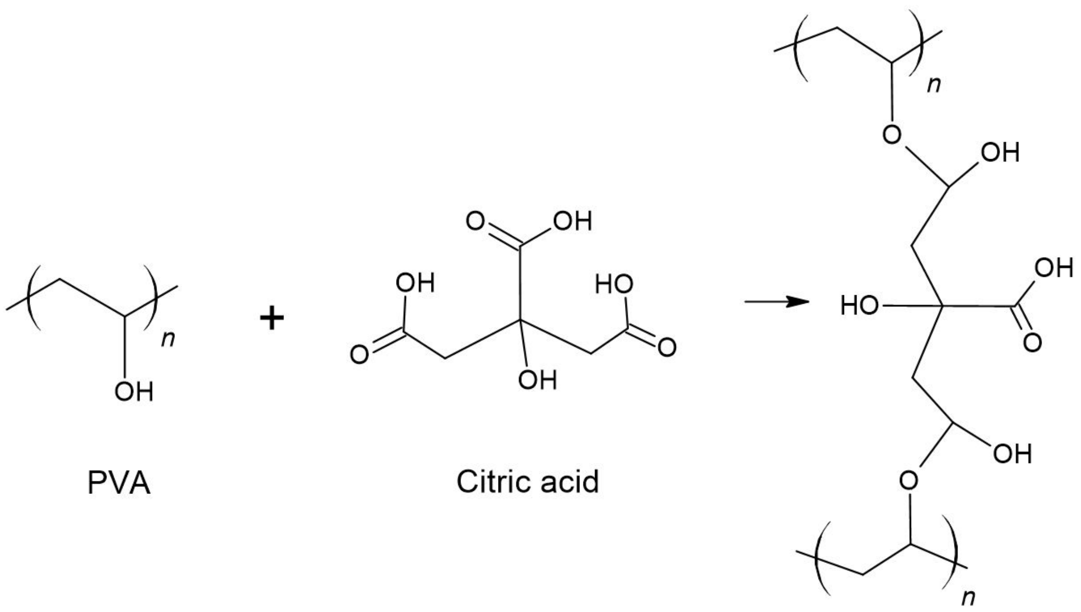

Fig. S2. Mechanism of esterification reaction occurring during crosslinking of PVA.

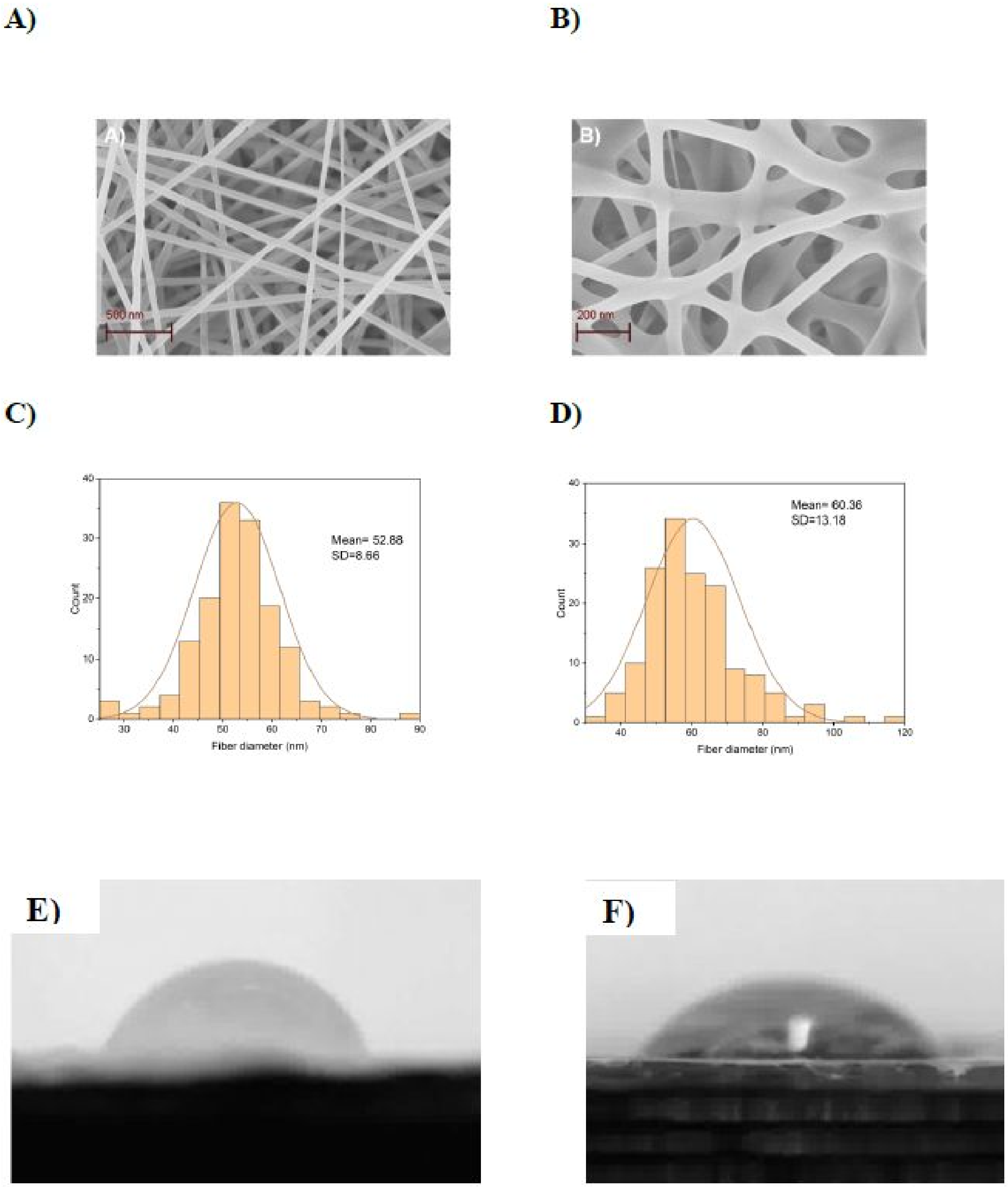

Fig. S3. SEM images and size distribution of PVA nanofibers A), C) before and B), D) after crosslinking and applying ion-selective cocktail, inset shows a nanofibers mat after crosslinking of PVA but prior cocktail application. Water contact angle measurements of crosslinked PVA nanofibers E) before and F) after applying a cocktail solution.

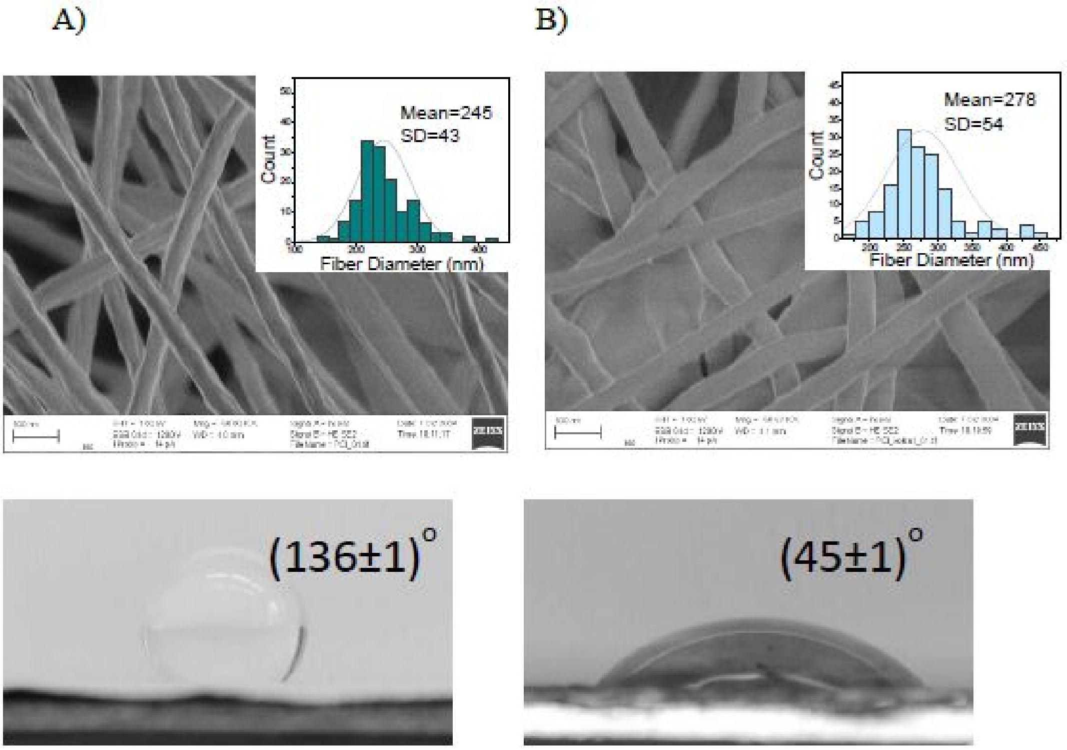

Fig. S4. SEM images of as obtained PCL nanofibers mat and water contact angle: A) as obtained mats, B) mats post application of PVA based micelle containing ATBC.

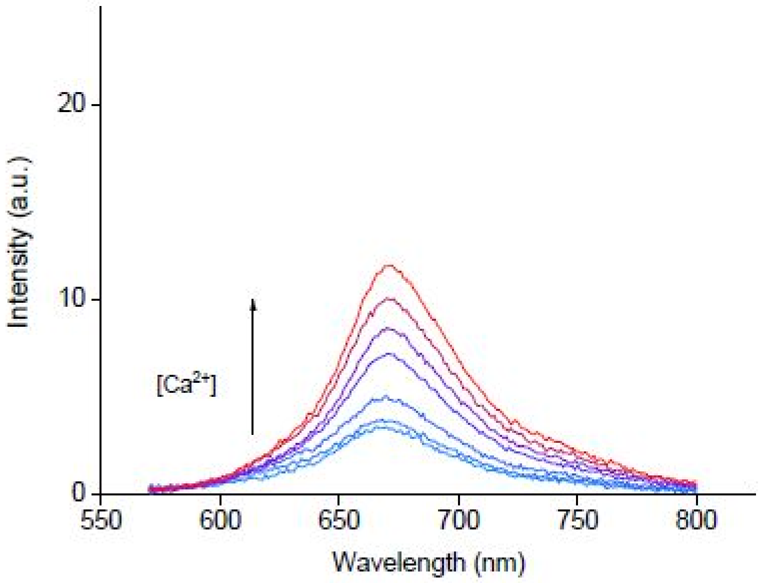

Fig. S5. Emission spectra of solution post removing Ca^{2+}-selective PCL mat holder, recorded for different analyte Ca^{2+} concentration, within the range from 10^{-5} to 10^{-1} M, spectra were recorded using excitation wavelength as in other experiment.

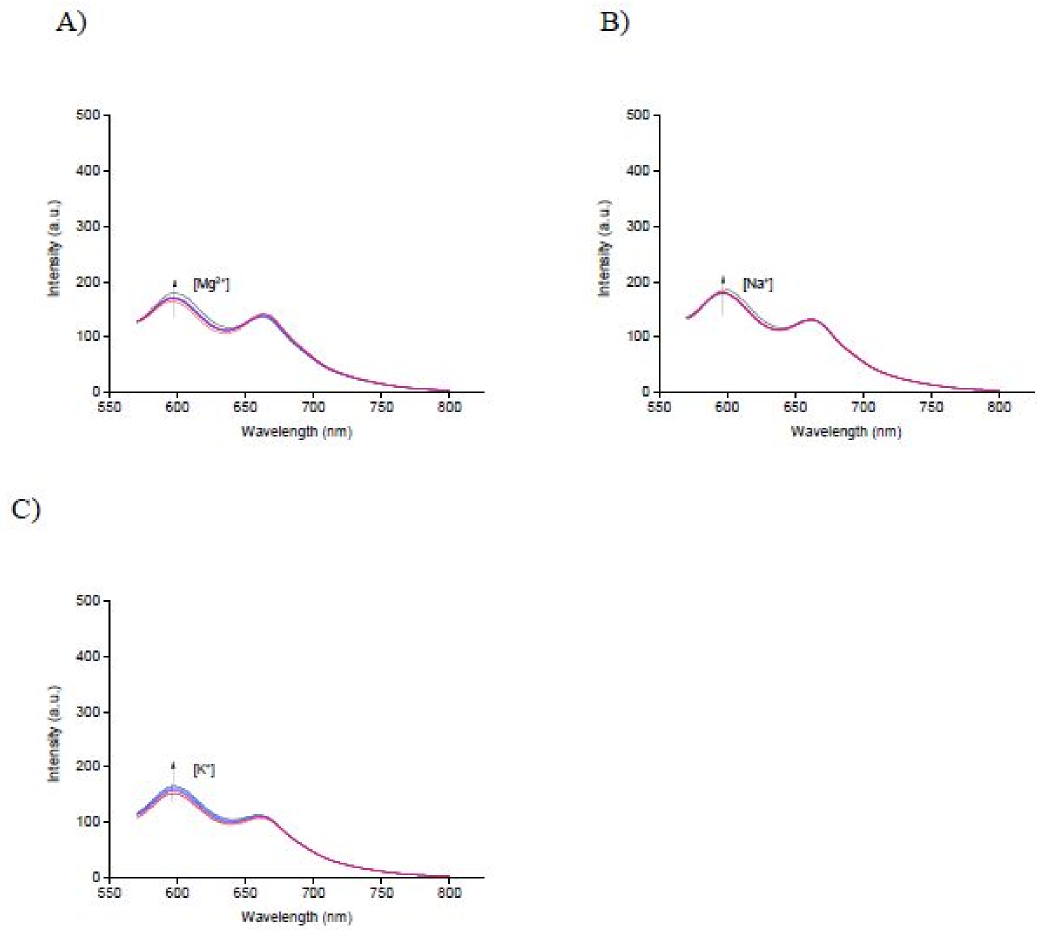

Fig. S6. Emission spectra of Ca^{2+}-selective PCL mat for increasing concentration of model interferents within the range from 10^{-6} to 10^{-1} M, recorded in unbuffered aqueous sample after 5 min probe – sample contact time, excitation wavelength 550 nm. A) Mg^{2+}, B) Na^{+}, C) K^{+} concentration changes.
